# 

**Published:** 1900-08

**Authors:** 


### PERSONALS.

Prof. R. S. Huidekoper, of Washington, D. C., with several nephews and nieces, spent two weeks in July camping along the historic Brandywine.

Dr. E. L. Cornman, of Gladwyne, Pa., of the class of 1900 of the Veterinary Department of the University of Pennsylvania, has recently become a member of Cassia Lodge, F. & A. M.

Dr. R. R. Dinwiddie, of Fayetteville, Ark., directs the work of the Laboratory of Zoöpathology and Bacteriology of the Arkansas Agricultural Experiment Station.

Veterinarian Edgar Odell, of New York City, is among the contributors to the *Rider and Driver* Mounted Police Medal and Hospital Fund.

Dr. W. W. Richards, of San Diego, Cal., is now Assistant City Veterinarian of Manila, Philippine Islands, attached to the City Board of Health.

Dr. David S. White, of the Veterinary Department of the Ohio State University, has been directed by the Board of Trustees to prepare plans for new buildings for the veterinary school.

Among Atlantic City's boardwalk visitors on Sunday were to be noted Drs. W. S. Kooker, of Philadelphia; James B. Rayner, of West Chester; B. M. Underhill, of Media, and A. T. Sellers, of Camden, N. J.

Dr. Charles F. Oat, of West Chester, Pa., is a lover of dogs, and finds life more pleasant on the farm when his morning slumbers are broken by the music of his canine friends.

Dr. L. A. Nolan, of Baltimore, Md., takes an active interest in Maryland politics. He says that State will return again in November to its first love and be found in the Democratic column.

"Ventilating a Stable" is the subject of a paper by Dr. E. A. A. Grange, in the *Rider and Driver* of August 11, 1900.

Drs. Pearson and Huidekoper had an interview recently with President McKinley in Washington, who expressed himself on the Army Bill, saying, "I am very much interested in this veterinary matter, and you have my best wishes for success."

Dr. M. H. Reynolds, of St. Anthony Park, Minn., who was recently called to the Deanship of the Veterinary Department of the Iowa Agricultural College, is a member of the Amerian Medical and Minnesota State Medical Association, and a frequent contributor to the programmes of these Associations.

Two elephants, one with a broken lumbar vertebra and the other with acute endocarditis, and sudden death from overwork, was the sore experience in the routine practice of Dr. Clement, of Baltimore, last month.

Dr. Leonard Pearson, State Veterinarian of Pennsylvania, spent some ten days of July in Kentucky. "Blue-grass" maidens were the primary attraction, and may account for the prolonged stay.

Dr. Austin Peters, of Boston, Mass., will act as temporary State Secretary of the American Veterinary Medical Association during the absence of State Secretary Pierce.

Dr. S. Stewart, of Kansas City, Kan., has proved a most efficient Secretary of the American Veterinary Medical Association, and continues to retain a very keen interest in the work.

Dr. William H. Kelly, of Albany, N. Y., fills the rôles of Secretary of the State Board of Veterinary Medical Examiners and Resident State Secretary of the American Veterinary Medical Association. He is also a very active member in legislative matters concerning his State Association.

Dr. R. S. Huidekoper will spend August in Winchester, Mass.

Dr. Frank Standen, of Philadelphia, whose health has been impaired for some time, is now journeying through Europe in search of relief.

Dr. E. M. Ranck, of Philadelphia, is among the cottagers at Anglesea, N. J., for the summer.

